# Virulence regulator AphB enhances *toxR *transcription in *Vibrio cholerae*

**DOI:** 10.1186/1471-2180-10-3

**Published:** 2010-01-06

**Authors:** Xiao Xu, Andrew M Stern, Zhi Liu, Biao Kan, Jun Zhu

**Affiliations:** 1 State Key Laboratory for Infectious Disease Prevention and Control, National Institute for Communicable Disease Control and Prevention, Beijing, PR China; 2 Department of Microbiology, University of Pennsylvania School of Medicine, Philadelphia, PA 19104; USA

## Abstract

**Background:**

*Vibrio cholerae *is the causative agent of cholera. Extensive studies reveal that complicated regulatory cascades regulate expression of virulence genes, the products of which are required for *V. cholerae *to colonize and cause disease. In this study, we investigated the expression of the key virulence regulator ToxR under different conditions.

**Results:**

We found that compared to that of wild type grown to stationary phase, the *toxR *expression was lower in an *aphB *mutant strain. AphB has been previously shown to be a key virulence regulator that is required to activate the expression of *tcpP*. When expressed constitutively, AphB is able to activate the *toxR *promoter. Furthermore, gel shift analysis indicates that AphB binds *toxR *promoter region directly. We also characterize the effect of AphB on the levels of the outer membrane porins OmpT and OmpU, which are known to be regulated by ToxR.

**Conclusions:**

Our data indicate that *V. cholerae *possesses an additional regulatory loop that use AphB to activate the expression of two virulence regulators, ToxR and TcpP, which together control the expression of the master virulence regulator ToxT.

## Background

**Figure 1:**
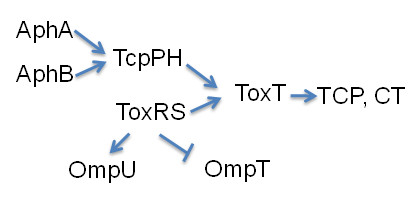
**The ToxR regulon**. AphA and AphB are known to activate *tcpPH *expression. TcpPH and ToxRS activate the expression of ToxT, which in turn activates the expression of the central virulence factors, cholera toxin (CT) and the toxin-coregulated pilus (TCP). ToxRS also upregulates OmpU and downregulates OmpT, which are outer membrane porins.

The Gram-negative bacterium *Vibrio cholerae *is the etiologic agent of cholera. The ability of *V. cholerae *to colonize and cause disease in hosts requires production of a number of virulence factors during infection. The two major virulence determinants of *V. cholerae *are encoded by two separate genetic elements: cholera toxin (CT), which causes the diarrhea characteristic of cholera, and the toxin-coregulated pilus (TCP), which is essential for attachment and colonization of intestinal epithelia [1,2]. CT is encoded by the *ctxAB *genes on the lysogenic CTXÖ bacteriophage [3]. The genes required for TCP synthesis and the genes encoding the virulence transcriptional activators ToxT and TcpP are located on a 40-kb *Vibrio *pathogenicity island (VPI) [4]. Coordinate expression of *V. cholerae *virulence genes results from the activity of a cascading system of regulatory factors [5] (Fig. 1).

The primary direct transcriptional activator of *V. cholerae *virulence genes, including *ctxAB *and *tcpA*, is ToxT, a member of the AraC family of proteins [6]. The expression of ToxT is under the control of a complex regulatory pathway. The ToxR protein was identified as the first positive regulator of *V. cholerae *virulence genes [7]. ToxR activity requires the presence of another protein, ToxS, which is also localized to the inner membrane, but is thought to reside predominantly in the periplasm, where ToxR and ToxS are hypothesized to interact. ToxS serves as a mediator of ToxR function, perhaps by influencing its stability and/or capacity to dimerize [6]. To regulate expression of *toxT*, ToxR acts in conjunction with a second transcriptional activator, TcpP, which is also membrane-localized with a cytoplasmic DNA-binding and other periplasmic domains [8]. TcpP, like ToxR, requires the presence of a membrane-bound effector protein, TcpH, which interacts with TcpP [9]. Two activators encoded by unlinked genes, AphA and AphB, regulate the transcription of *tcpPH*. AphA is a dimer with an N-terminal winged-helix DNA binding domain that is structurally similar to those of MarR family transcriptional regulators [10]. AphA cannot activate transcription of *tcpP *alone, but requires interaction with the LysR-type regulator AphB that binds downstream of the AphA binding site [11].

The ToxR and ToxS regulatory proteins have long been considered to be at the root of the *V. cholerae *virulence regulon, called the ToxR regulon. The membrane localization of ToxR suggests that it may directly sense and respond to environmental signals such as temperature, osmolarity, and pH [12]. In addition to regulating the expression *toxT*, ToxR activates the transcription of *ompU *and represses the transcription of *ompT*, outer membrane porins important for *V. cholerae *virulence [13,14]. Microarray analysis indicates that ToxR regulates additional genes, including a large number of genes involved in cellular transport, energy metabolism, motility, and iron uptake [15]. It has been reported that levels of ToxR protein appear to remain constant under various *in vitro *conditions [16,17] and are modulated by the heat shock response [18].

To further investigate the relationship between *toxR *expression and other virulence regulators, we analyzed *toxR *transcription and ToxR protein levels in various virulence regulator mutants. We found that in addition to activating *tcpP*, AphB was required for full expression of ToxR in *V. cholerae *stationary growth phase. AphB regulated *toxR *directly as purified recombinant AphB binds to the *toxR *promoter. This study suggests that *V. cholerae *may use this additional layer of activation to turn on virulence factor production efficiently in optimal conditions.

## Results and Discussion

### Examination of *toxR *expression under different *in vitro *conditions using a transcriptional fusion reporter

**Figure 2:**
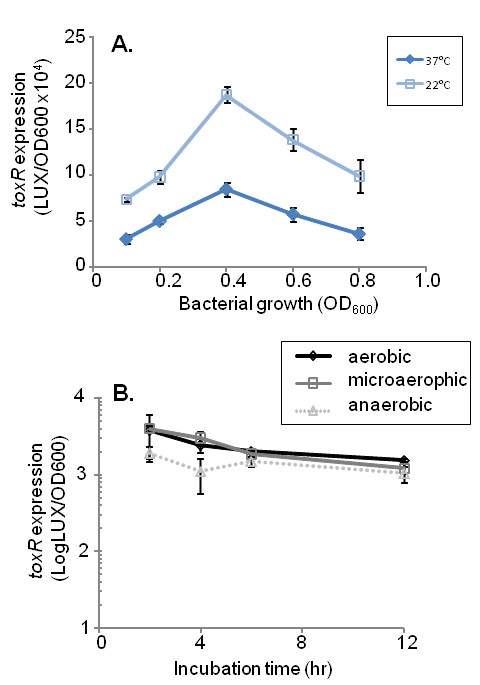
**The expression of *toxR *in wild type under different conditions using a P_*toxR*_*-luxCDABE *transcriptional reporter**. (A). The reporter strain was grown at 22°C or 37°C, and at successive time points, luminescence was measured. Units are arbitrary light units/OD_600_. The results are the average of three experiments ± SD. (B). The reporter strain was grown at 37°C aerobically, in an anaerobic chamber (Mini MACS Anaerobic workstation, Microbiology International) or in a BBL CampyPak Microaerophilic System. At different time points, samples were withdrawn and luminescence was measured. Units are arbitrary light units/OD_600_. The results are the average of three experiments ± SD.

ToxR is one of two proteins, along with TcpP, shown to activate the expression of ToxT, the master virulence activator in *V. cholerae *(Fig. 1). The expression of *tcpP *has been shown to be induced by AphA and AphB [11,19], while *toxR *has been thought to be constitutively expressed and only modulated by temperature [16,18]. To measure *toxR *expression, we placed the *toxR *promoter upstream of the *luxCDABE *operon on a plasmid [20] and transformed into wild type *V. cholerae*. We then grew the resulting cells at 37°C or 22°C. Expression of *P*_*toxR*_*-luxCDABE *was significantly increased at 22°C (Fig. 2A), consistent with the previous report [18] that the expression of *toxR *is modulated by temperatures. Since the availability of oxygen concentrations is different during *V. cholerae *infection, we also examined the expression of *toxR *under varying oxygen concentrations (Fig. 2B). The *lux *expression was similar under each condition, suggesting that oxygen levels do not regulate *toxR *expression.

### Influence of virulence regulatory proteins on *toxR *expression

**Figure 3:**
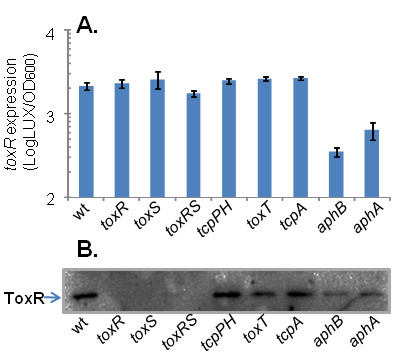
**Expression of *toxR *in different mutations of *V. cholerae***. (A) Activity of P_*toxR*_*-luxCDABE *reporter constructs (blue bars) in *V. cholerae *wild type and virulence regulatory mutants. Cultures were grown at 37°C overnight. Units are arbitrary light units/OD_600_. The results are the average of three experiments ± SD. (B) Analysis of samples in (A) by Western blot with anti-ToxR antiserum.

To investigate molecular influences on *toxR *expression, we introduced the *P*_*toxR*_*-lux *construct into various strains of *V. cholerae *with mutations in virulence regulator genes. We also included a *tcpA *mutant because a previous study showed that TcpA, the major subunit of TCP pilin [2], affects cholera toxin gene expression *in vivo *but not *in vitro *[21]. We grew these strains at 37°C for 12 hours and measured luminescence (Fig. 3A). We found that ToxR and ToxS did not affect *toxR *expression, indicating that ToxR does not autoregulate. The expression of *toxR *in *tcpPH*, *toxT*, and *tcpA *mutants remained the same as that of wild type, but it was significantly decreased in *aphA *and *aphB *mutant strains (approximately 3- and 6-fold, respectively). Of note, *toxR *expression in wild type and *aphA *or *aphB *mutants remained similar in the early and logarithmic phases of growth (data not shown). We also examined *toxR *expression in wild type and various virulence regulatory mutants grown under the AKI condition [22], in which virulence genes are induced in El Tor strains of *V. cholerae*. We found that *toxR *expression was decreased in both *aphA *and *aphB *mutants to a similar degree as those grown in LB medium (data not shown). These data suggest that AphA and AphB may be important factors in increasing *toxR *expression during *V. cholerae *stationary growth. These studies were confirmed by Western blot to examine ToxR protein levels (Fig. 3B): compared to those of wild type and other mutant strains, ToxR protein levels were notably decreased in the *aphA *and *aphB *mutants. Interestingly, while *toxR *transcription was unchanged in *toxS *mutant (Fig. 3A), ToxR proteins were not detected in the absence of ToxS, suggesting that the ToxR effector ToxS may affect ToxR stability, at least in the stationary phase condition we tested. Beck *et al*. reported that loss of ToxS had no measurable negative effect on steady-state levels of the ToxR protein at the mid-log phase growth [9]. The decreased ToxR expression at stationary phase in a *toxS *mutant is the subject of another investigation.

### AphB directly regulates *toxR *expression

**Figure 4:**
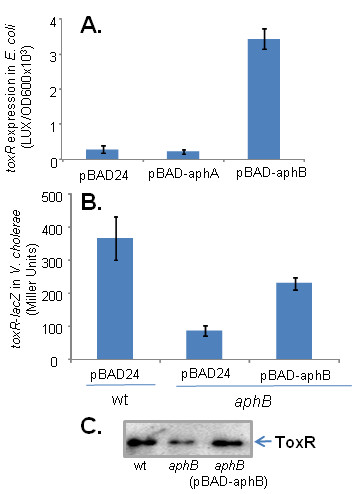
**Expression of *toxR *in the presence of AphA or AphB**. (A). Activity of P_*toxR*_*-luxCDABE *reporter constructs (blue bars) in *E. coli *containing pBAD24 as a vector control, pBAD-*aphA *or pBAD-*aphB*. Arabinose (0.01%) was used to induce P_*BAD *_promoters and cultures were grown at 37°C to stationary phase. Units are arbitrary light units/OD_600_. The results are the average of three experiments ± SD. (B). *toxR-lacZ *expression (blue bars). *V. cholerae lacZ*^- ^strains containing *toxR-lacZ *chromosomal transcriptional fusions and either pBAD24 or pBAD-aphB were grown in LB containing 0.01% arabinose at 37°C for 12 hrs and β-galactosidase activities of the cultures were measured [35] and reported as the Miller Unit. The results are the average of three experiments ± SD. (C). Analysis of samples in (B) by Western blot with anti-ToxR antiserum.

Knowing that full expression of *ToxR *required both AphA and AphB, we sought to determine which was directly responsible for this effect. To this end, we placed *aphA *and *aphB *under control of an arabinose-inducible promoter and measured its effect on *P*_*toxR*_*-luxCDABE *transcription in *E. coli*. Overexpression of AphB, but not AphA, dramatically increased *toxR *transcription (Fig. 4A). We currently do not know why in *V. cholerae*, both AphA and AphB are required to fully activate *toxR *expression, while in *E. coli*, only AphB can induce *P*_*toxR*_*-luxCDABE*. One possibility is that in *V. cholerae*, the expression of *aphB *is dependent on AphA. However, we examined *aphB *expression in wild type and *aphA *mutant strains and did not detect any difference. Another possibility is that AphA may indirectly activate ToxR expression through an intermediate which is absent in *E. coli*, or that AphA is required to repress an inhibitor of AphB that is present in *V. cholera *but not in *E. coli*. AphA has been shown to regulate a number of other genes [23,24]. The activation of ToxR hinted at in this study may thus rely on the regulation of other members of the regulation cascade not yet elucidated. We further confirmed AphB regulation of *toxR *in *V. cholerae *using a chromosomal transcriptional *toxR-lacZ *fusion (Fig. 4B). We found that compared to that of wild type, *toxR-lacZ *expression was reduced in *aphB *mutants, while expression of *aphB *from a plasmid in this mutant restored *toxR *expression (Fig. 4B) and ToxR production (Fig. 4C).

**Figure 5:**
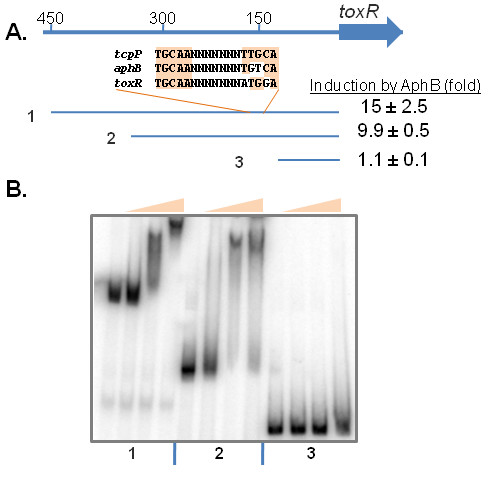
**AphB binds to the *toxR *promoter region to regulate *toxR *gene expression**. (A) Three different lengths of *toxR *promoter regions used in (B) were PCR amplified and cloned into pBBRlux containing a transcriptional *lux *reporter. The level of Lux induction with pBAD-*aphB *compared to pBAD24 in *E. coli *in the presence of 0.01% arabinose is given in the table. Alignment of putative AphB binding sites in *tcpP*, *aphB*, and *toxR *promoter region is given. (B) Gel shift assays using purified MBP-AphB and DNA containing various lengths of the regulatory regions of the *toxR *promoter. Protein concentrations used in the gel shift assay (shown as shaded triangles) were 0, 20, 40, 80 ng/reaction (5 μl).

To investigate whether AphB-mediated activation of *toxR *is direct or acts through another regulator present in *E. coli*, we purified AphB as an MBP (maltose-binding protein) fusion. Recombinant AphB is functional, as it could activate *tcpP *transcription in *E. coli *(data not shown). We then performed Electrophoretic Mobility Shift Assays (EMSA) using MBP-AphB and various lengths of *toxR *promoter DNA (Fig. 5A). Fig. 5B shows that purified MBP-AphB was able to shift the two large *toxR *promoter fragments. All of these mobility shifts could be inhibited by the addition of unlabeled specific DNA, indicating that the binding of AphB to these DNA sequences is specific (data not shown). AphB was unable to shift the shortest *toxR *promoter fragment containing the 130 base pairs closest to the *toxR *translational start site, suggesting that the AphB binding site is located between 130 and 450 base pairs upstream of the *toxR *gene. It has been reported that AphB binds and regulates *tcpP *and *aphB *promoter regions, and the AphB recognition sites in these promoters were identified [25]. We identified a similar putative AphB binding site in the *toxR *promoter region approximately 150 bp upstream of the *toxR *translational start (Fig. 5). Further studies are required to test whether AphB protein binds this putative recognition site. Consistent with the gel shift data, AphB could not induce *toxR *expression when the 130-bp fragment was fused with the *luxCDABE *reporter in *E. coli *(Fig. 5A). Taken together, these data suggest that AphB directly regulates *toxR *expression.

### The effects of AphB on ToxR-regulated genes

**Figure 6:**
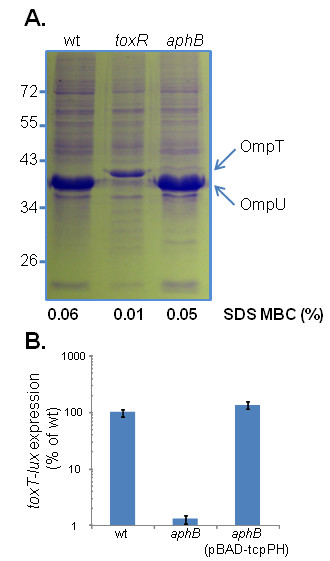
**The influence of AphB on *V. cholerae *outer membrane composition, SDS resistance, and *toxT *expression**. (A). Analysis of outer membrane preparations of *V. cholerae *derivatives. SDS-PAGE gel stained with Coomassie blue. OmpT and OmpU are indicated at the right. The minimum bactericidal concentration (MBC) of SDS is listed below the SDS-PAGE gel. (B). Wild type or *aphB *mutant containing a P_*toxT*_-*luxCDABE *reporter plasmid with or without pBAD-tcpPH were grown under the AKI condition. 0.01% arabinose was added to induce P_*BAD*_-*tcpPH*. Lux expression (blue bars) was measured and normalized against *toxT *expression in wild type. The results are the average of three experiments ± SD.

In addition to regulation of *toxT*, ToxR has been previously shown to alter the porin levels in *V. cholerae *by activating expression of *ompU *and repressing *ompT *[26,27]. Since we showed that AphB affects ToxR levels, we hypothesized that AphB might thus indirectly modulate the expression of *ompU *and *ompT *as well. We performed SDS-PAGE on total protein extracts of wild type *V. cholerae *as well as *toxR *and *aphB *mutants. As expected, the *toxR *strain had significantly lower OmpU and higher OmpT levels than in the wild-type strain. Interestingly, the *aphB *mutant strain produced slightly higher levels of OmpT than wild type, though OmpU levels did not seem to change (Fig. 6A). In addition, Provenzano et al. showed that ToxR-dependent modulation of outer membrane proteins enhances *V. cholerae *resistance to antimicrobial compounds such as bile salts and sodium dodecyl sulfate (SDS) [28]. We confirmed that the *toxR *mutant strain had a reduced minimum bactericidal concentration (MBC) of SDS compared to wild type strains, but AphB did not affect the MBC of SDS (Fig. 6A). Thus, AphB may only subtly modulate outer membrane porin expression through its effect on *toxR *expression. This may be another downstream effect of AphB on the virulence capabilities of *V. cholerae *in addition to its better characterized influences on ToxT levels. Moreover, as both ToxR and TcpP are required to activate *toxT *expression and AphB is required to activate *tcpP *expression (Fig. 1) [19,29], we tested whether AphB effects on *toxR *expression affect *toxT *expression under the AKI virulence induction condition [22]. As expected, *toxT *expression in *aphB *mutants was significantly reduced as compared to that of wild type (Fig. 6B), however, bypassing the AphB regulation of *tcpP *by constitutively expressing *tcpPH *(pBAD-tcpPH induced with 0.01% arabinose) restored *toxT *expression in *aphB *mutants. These data suggest that AphB modulation of *toxR *expression has minor effects on virulence gene expression as compared to that of AphB regulation of *tcpP *under the condition we tested.

## Conclusion

The ToxR regulon is the classic virulence gene regulation pathway in *V. cholerae*. In this pathway, AphA and AphB activate *tcpP *transcriptional expression directly by binding to different promoter regions of *tcpP*. ToxR and TcpP cooperate in turn by binding different sites of the *toxT *promoter to activate transcription, leading to the production of the virulence factors TCP and CT. However, the full ToxR regulon is more complex than previously thought. In this paper, we showed that AphA and AphB are also necessary for full ToxR production at the stationary phase. Furthermore, we demonstrated that AphB is sufficient for *toxR *transcriptional activation in the heterogenic host *E. coli *through binding of the toxR promoter region. Thus, the effect of AphB on ToxR levels propagates further in the transcription cascade, increasing the transcription of a key gene in *V. cholerae *pathogenesis, *toxT*. We have therefore identified another factor responsible for altering end product levels in the *V. cholerae *virulence axis. Since AphB is at the top of a virulence cascade with multiple end pathways, it appears now that AphB is a central factor in switching the cell from an environmental state to a virulent one. Since it activates ToxR in addition to TcpP, and further influences porin expression, AphB is a divergence point at which nonlinearity is introduced into the *V. cholerae *virulence pathway. Eukaryotic cells have extremely complex networks of protein and DNA interactions leading to precise control of protein expression levels. Having a more complex network of transcriptional activation and repression in the *V. cholerae *virulence cascade could enable the bacterial cell to fine-tune its expression levels to optimize its ability to colonize the intestine and spread to other hosts.

## Methods

### Bacterial strains, plasmids and media

All experiments were performed with El Tor *Vibrio cholerae *C6706 [30] or *Escherichia coli *DH5α, which were grown in LB with relevant antibiotics at 37°C, except where noted. *V. cholerae *virulence genes were induced *in vitro *(the AKI condition) as previously described [22]. Briefly, 3 ml of AKI medium was inoculated with 0.5 μl of overnight culture and incubated for 4 hrs at 37°C without agitation. 1 ml of culture was transferred to a fresh tube and incubated with shaking for a further 4 hrs at 37°C.

*P*_*toxR*_*-luxCDABE *fusion plasmid was constructed by polymerase chain reaction (PCR) amplifying the *toxR *promoter regions, ranging from 450 bp, 300 bp, to 130 bp, respectively, and cloning them into the pBBRlux vector [20]. P_*toxT*_-*luxCDABE *plasmid was constructed by cloning *toxT *promoter regions into the pBBRlux vector. The chromosomal *toxR-lacZ *transcriptional fusion was constructed by cloning the 5' *toxR *region into the suicide vector pVIK112, which also contains a promoterless *lacZ *gene [31]. The resulting plasmid was then integrated into the chromosomes of *V. cholerae lacZ*^- ^strains by homologous recombination to create a single-copy *toxR-lacZ *and an intact copy of *toxR*. P_*BAD*_-controlled *aphA *and *aphB *plasmids were constructed by cloning *aphA *and *aphB *coding sequences into the pBAD24 vector [32]. pBAD-tcpPH plasmid construct was described in [8]. In-frame deletions of *toxR, toxS, tcpP, tcpA, toxT, aphA*, and *aphB *were either described previously [15] or constructed by cloning the regions flanking target genes into the suicide vector pWM91 containing a *sacB *counter-selectable marker [33]. The resulting plasmids were introduced into *V. cholerae *by conjugation and deletion mutants were selected for double homologous recombination events.

### Lux activity assays

Bacteria were grown at 37°C or 22°C under conditions indicated. At different time points, cultures were withdrawn and luminescence was measured by using a Bio-Tek Synergy HT spectrophotometer. Lux expression is calculated as light units/OD_600_.

### Western blotting and SDS-PAGE electrophoresis

Whole-cell lysates were prepared from bacteria overnight cultures in LB conditions at 37°C and samples were normalized to the amount of total protein as assayed by the Biorad protein assay (Biorad). The isolation of outer membrane (OM) proteins from *V. cholerae *was performed using the method described by Miller and Mekalanos [34]. Whole-cell lysates or OM preparations were separated by sodium dodecyl sulfate-polyacrylamide gel electrophoresis (SDS-PAGE) on a 10% polyacrylamide gel and stained with Coomassie brilliant blue for visualization. SDS-PAGE gels were transferred to nitrocellulose membrane for Western blot analysis using polyclonal rabbit anti-ToxR antibody.

### Gel retardation assays

MBP-AphB protein was purified through amylose columns according to the manufacturer's instructions (New England Biolabs). PCR products of the different lengths of *toxR *promoter regions were digested with EcoRI and end-labeled using [α-^32^P]dATP and the Klenow fragment of DNA polymerase I. Binding reactions contained 0.1 ng of DNA and MBP-AphB proteins in a buffer consisting of 10 mM Tris-HCl (pH 7.9), 1 mM EDTA, 1 mM dithiothreitol, 60 mM KCl, and 30 mg of calf thymus DNA/ml. After 20 minutes of incubation at 25°C, samples were size-fractionated using 5% polyacrylamide gels in 1× TAE buffer (40 mM Tris-acetate, 2 mM EDTA; pH 8.5). The radioactivity of free DNA and AphB-DNA complexes was visualized by using a Typhoon 9410 PhosphorImager (Molecular Dynamics).

## Authors' contributions

XX, AS, ZL, BK, and JZ designed research; XX, AS, and ZL performed research; XX, AS, and JZ analyzed data, XX, AS, ZL, BK, and JZ wrote the paper. All authors read and approved the final manuscript.
